# Care provided by humanoid robots: a scoping review

**DOI:** 10.17533/udea.iee.v43n1e11

**Published:** 2025-04-28

**Authors:** Lailla Ketly Ferreira Tiradentes Ruiz, Tatiana da Silva Melo Malaquias, Geraldo Bezerra, Isabel Cristina Kowal Olm Cunha, Rosangela Aparecida Pimenta, Patrícia Aroni Dadalt, Maria do Carmo Fernandez Lourenço Haddad

**Affiliations:** 1 Nurse, Ph.D. Email: laillaftruiz@gmail.com. Corresponding author. https://orcid.org/0000-0003-2650-2255 Universidade Estadual de Londrina Brazil laillaftruiz@gmail.com; 2 Nurse, Ph.D. Email: tatieangel@yahoo.com.br. https://orcid.org/0000-0001-5541-441X Universidade Estadual do Oeste do Paraná Brazil tatieangel@yahoo.com.br; 3 Physician, Ph.D. Email: geraldobezerrajr@unifor.br. https://orcid.org/0000-0002-8971-0994 Universidade de Fortaleza Brazil geraldobezerrajr@unifor.br; 4 Nurse, Ph.D. Email: isabelcunha@unifesp.br. https://orcid.org/0000-0001-6374-5665 Universidade Federal de São Paulo Brazil isabelcunha@unifesp.br; 5 Nurse, Ph.D, Email: ropimentaferrari@uel.br https://orcid.org/0000-0003-0157-7461 Universidade Estadual de Londrina Brazil ropimentaferrari@uel.br; 6 Nurse, Ph.D. Email: patriciaaroni@uel.br. https://orcid.org/0000-0001-5092-2714 Universidade Estadual de Londrina Brazil patriciaaroni@uel.br; 7 Nurse, Ph.D. Email: mhaddad@uel.br. https://orcid.org/0000-0001-7564-8563 Universidade Estadual de Londrina Brazil mhaddad@uel.br; 8 State University of Londrina (UEL), Londrina, Paraná, Brazil. Universidade Estadual de Londrina State University of Londrina (UEL) Londrina Paraná Brazil; 9 State University of Centro Oeste (UNICENTRO), Guarapuava, Paraná, Brazil Universidade Estadual do Oeste do Paraná State University of Centro Oeste (UNICENTRO) Guarapuava Paraná Brazil; 10 University of Fortaleza (UNIFOR), Fortaleza, Ceará, Brazil Universidade de Fortaleza University of Fortaleza (UNIFOR) Fortaleza Ceará Brazil; 11 Federal University of São Paulo (UNIFESP), São Paulo, São Paulo, Brazil Universidade Federal de São Paulo Federal University of São Paulo (UNIFESP) São Paulo São Paulo Brazil

**Keywords:** Robotics, artificial intelligence, patient care, hospital care, ambulatory care, scoping review., Robótica, inteligencia artificial, atención al paciente, atención hospitalaria, atención ambulatoria, revisión., robôs humanoides, inteligência artificial, assistência ao paciente, assistência hospitalar, assistência ambulatorial, revisão de escopo.

## Abstract

**Objective.:**

To identify the evidence in the literature regarding the care provided to the population by humanoid robots.

**Methods.:**

A scoping review based on the guidelines established by the Joanna Briggs Institute. The Preferred Reporting Items for Scoping Review (PRISMA-ScR) checklist was followed. The review protocol was registered on the Open Science Framework under the number osf.io/6ur93. The search was conducted in November 2023 in the following databases: PubMed®, EMBASE®, LILACS, Web of Science, Scopus®, and CINAHL, as well as in the gray literature, including Google Scholar and the Catalog of Theses and Dissertations of the Coordination for the Improvement of Higher Education Personnel (CAPES), using the search strategy: “humanoid robot*” AND “patient*”.

**Results.:**

A total of 27 articles were analyzed. Most of the identified studies were conducted in hospital settings (*n*=13), with a primary focus on adults (*n*=10) and children (*n*=8). The countries with the highest number of publications were Japan (*n*=6), Canada (*n*=5), and France (*n*=4). Three areas of care were identified: social interaction (*n*=17), physical rehabilitation (*n*=7), and dissemination of health information (*n*=3). Additionally, only four studies involved collaboration between humanoid robots and healthcare providers.

**Conclusion.:**

Despite the increasing use of humanoid robots in healthcare, it remains essential to enhance their integration with professionals in the field. Social interaction highlighted the need to improve patient care, underscoring the importance of aligning the capabilities of these robots with the expertise of healthcare providers. Accordingly, future research should focus on developing strategies that ensure this technology not only assists but also optimizes the quality of care and strengthens interdisciplinary collaboration.

## Resumen

**Objetivo.:**

Identificar la evidencia existente en la literatura científica sobre los cuiadados asistenciales prestados a la población por robots humanoides.

**Métododos.:**

Revisión de alcance basada en las directrices establecidas por el Instituto Joanna Briggs. Se siguió la lista de verificación Preferred Reporting Items for Scoping Review (PRISMA-ScR). El protocolo de revisión se registró en el Open Science Framework con el número: osf.io/6ur93. La búsqueda se realizó en las siguientes bases de datos: Pubmed®, EMBASE®, LILACS, Web of Science, Scopus® y CINAH, y en literatura gris, Google Scholar y el Catálogo de Tesis y Disertaciones de la Coordinación para la Mejora del Personal de Educación Superior (CAPES), utilizando como estrategia de búsqueda: «humanoid robot*» AND «patient*».

**Resultados.:**

Se analizaron 27 artículos. La mayoría de los estudios identificados se realizaron en entornos hospitalarios (*n*=13), y se centraron principalmente en adultos (*n*=10) y niños (*n*=8). Los países que ocuparon un lugar destacado en las publicaciones fueron Japón (*n*=6), Canadá (n=5) y Francia (*n*=4). Se identificaron tres áreas de atención: interacción social (*n*=17), rehabilitación física (*n*=7) y difusión de información sanitaria (*n*=3); solamente se identificaron cuatro estudios que implicaban la colaboración entre robots humanoides y profesionales sanitarios.

**Conclusión.:**

A pesar del creciente uso de robots humanoides en la asistencia sanitaria, sigue siendo esencial mejorar su integración con los profesionales sanitarios. La interacción social reveló la necesidad de mejorar la atención al paciente, lo que pone de manifiesto la importancia de alinear las capacidades de estos robots con los conocimientos de los profesionales sanitarios. Por tanto, la investigación futura debe centrarse en desarrollar estrategias para garantizar que esta tecnología no solamente ayude, sino que también optimice la calidad de la asistencia y refuerce la colaboración interdisciplinar.

## Resumo

**Objetivo.:**

Identificar as evidências na literatura quanto aos cuidados assistenciais prestados à população por robôs humanoides.

**Métodos.:**

Revisão de escopo embasada nas diretrizes estabelecidas pelo Joanna Briggs Institute. Seguiu-se o *checklist* do *Preferred Reporting Items for Scoping Review (PRISMA-ScR).* O protocolo da revisão foi registrado na *Open Science Framework* com o número osf.io/6ur93. A busca ocorreu em novembro de 2023 nas bases de dados: *Pubmed®, EMBASE®, LILACS, Web of Science, Scopus® e CINAH*, e na literatura cinzenta*, Google Scholar* e Catálogo de teses e dissertação da Coordenação de Aperfeiçoamento de Pessoal de Nível Superior (CAPES), sendo utilizada como estratégia de busca: “humanoid robot*” AND “patient*”.

**Resultados.:**

Foram analisados 27 artigos. A maioria dos estudos identificados foi em ambientes hospitalares (*n*=13), com foco principal em adultos (*n*=10), e crianças (*n*=8). Os países que tiveram destaque nas publicações foram Japão (*n*=6), Canadá (*n*=5) e França (*n*=4). Foram identificadas três áreas de cuidados: interação social (*n*=17), reabilitação física (*n*=7), e disseminação de informações em saúde (*n*=3); além disso, foram identificados apenas quatro estudos que envolveram a colaboração entre robôs humanoides e profissionais de saúde.

**Conclusão.:**

A pesar do crescente uso de robôs humanoides na área da saúde, ainda é essencial aprimorar sua integração com os profissionais da área. A interação social revelou a necessidade de melhorar a atenção ao paciente, evidenciando a importância de um alinhamento entre as capacidades desses robôs e o conhecimento dos profissionais de saúde. Dessa forma, pesquisas futuras, devem focar no desenvolvimento de estratégias que garantam que essa tecnologia não apenas auxilie, mas também otimize a qualidade do atendimento e fortaleça a colaboração interdisciplinar.

## Introduction

Technology in the healthcare sector has been gaining significant and gradual prominence since the Industrial Revolution, marked by sophisticated equipment, particularly for diagnostic purposes.^1^ The global landscape presents major challenges concerning population health, requiring innovative and effective actions from technology industries focused on this field, considering that the global population is aging. It is estimated that by 2050, the world population over 60 years of age will reach approximately two billion, an increase of 900 million compared to 2015. People are living longer, however, they will be susceptible to chronic conditions that often lead to a dependence on care. In parallel with this context, there is a shortage of healthcare providers, which places enormous pressure on healthcare systems.^2^

Various technological tools play a crucial role in enhancing communication, productivity, and information management. The adoption of digital tools, such as videoconferencing platforms, has transformed work processes, leading to greater efficiency and cost savings. Consequently, the corporate world is under pressure to adopt technology to remain competitive in an evolving business environment.^3^ Given this, it becomes necessary to reconsider population health, where innovative measures reshape the care process. In light of this concern, initiatives focused on technological innovations and artificial intelligence (AI) emerge as important tools to support care provision.^4^ However, the integration of technological advancements into healthcare is not entirely new. A significant milestone occurred in the 1990s with the advent of robotic surgery, which enabled surgical procedures to be performed with greater detail and precision.^5^

More recently, the COVID-19 pandemic further reinforced the drive for technological adoption in healthcare, as there was a need to change the traditional care model.^6^ As a result, global healthcare systems seek effective tools to combat diseases while also aiming for faster, more modern, and preferably cost-effective processes.^7^ In this context, AI has the capability to approximate technology to human cognition, offering reasoning, strategies, speech, and even support for daily tasks.^4^ Advancements in this area have led to the emergence of a bipedal robot with a humanoid appearance, known as a humanoid robot (HR), playing a significant role in improving the quality of life of individuals with physical or mental impairments.^8^ This type of technology has been studied to facilitate healthcare-related activities. Additionally, there is growing concern in nursing regarding the alleviation of physical strain during bedside patient care, which is a leading cause of occupational illnesses and absences in this professional group.^9^^,^^10^

In Brazil, HRs have been utilized in various sectors, demonstrating their potential in multiple applications. Pilot studies have also explored the use of robots as receptionists in public offices, assisting visitors with navigation and inquiries.^11^ Furthermore, HRs have been deployed in shopping centers, where they guide and provide information to the public. In an innovative initiative, a municipality in southern Brazil introduced a robot into the daily activities of a clinic for autistic patients. This Brazilian-made robot was initially designed for reception, training, and event-related functions.^12^ Another example of innovation is a Brazilian technology hub that has developed HRs for various roles, including customer service in stores and supermarkets, advertising, events, and transporting supplies in laboratories.^13^

It is important to address the concept of robotic technology specifically applied to healthcare, which includes robots for telemedicine, remote hospital visits, and home care support for geriatric or post-hospitalization patients with other clinical conditions.^14^ Although some of these robots do not strictly follow the humanoid model seen in international studies, Brazil has been investing in and recognizing the potential of this technology. These initiatives collectively highlight the diverse roles HRs play in Brazil, spanning healthcare, industrial environments, and attending the public. Accordingly, conducting a scoping review is justified to gather scientific evidence on this highly relevant topic. Therefore, this study aimed to identify the evidence in the literature regarding the care provided to the population by humanoid robots and how robotics is being applied in clinical practice, potentially having a direct impact on reducing healthcare system costs.

## Methods

This study was a scoping review (SR), guided by the principles outlined in the Joanna Briggs Institute (JBI) manuals^15^^,^^16^ and written following the Preferred Reporting Items for Scoping Reviews (PRISMA ScR).^17^^,^^18^ The SR protocol was registered on the Open Science Framework (OSF) online platform under the number osf.io/6ur93 in December 2021. The database search was conducted in two phases: the first in August 2022, aimed at identifying studies on nursing practice that addressed care actions directed at assisting patients with diseases. This search revealed discrepancies in the types of care described, extending beyond the initially defined concept (search 1).

After conceptual adjustments among the reviewers, in which the results were explored and the scope and diversity of the care identified in the first search were analyzed, a new search was conducted in November 2023 using the same databases and search strategy, designated as search 2. In this phase, the concept of care was broadened to include any human-robot interaction intended to replace actions previously performed by healthcare providers, focusing on promoting well-being and patient care. The emphasis shifted to the role of humanoid robots (HRs) in healthcare delivery. The results of this search (search 2) provide the findings and are discussed throughout this article.

*Eligibility criteria.* The PCC (Population, Concept, and Context) acronym was used, based on the research question. The population comprised human beings, regardless of age, who received care provided by humanoid robots. The concept considered studies depicting care practices performed by bipedal robots with human-like characteristics. The context included studies conducted in any care settings, such as public and private hospitals, clinics, nursing homes, older adult care facilities, home environments, and others. The guiding question for this review was: "What care services are provided to the population by humanoid robots?" Studies that answered the research question were considered eligible, regardless of the publication period or language. Studies where the target population was not human and those focused on technical and operational aspects of robots were excluded.

*Search strategy.* The search was developed using key terms found in the Health Sciences Descriptors (DeCS) and the MeSH Database, combined with the Boolean operators "AND" and "OR" in the following electronic databases: PubMed®, Excerpta Medical Database (EMBASE®), Latin American and Caribbean Health Sciences Literature (LILACS), Web of Science, Scopus®, and CINAHL. Gray literature searches were also conducted using Google Scholar and the Coordination for the Improvement of Higher Education Personnel (CAPES) catalog of theses and dissertations. An initial search was performed in the PubMed® and Scopus® databases, defining the search strategy as: "humanoid robot" AND "patient*", adapted for each database. This stage was conducted with the support of a librarian from a public university in the state of São Paulo.

*Study selection.* All types of studies, case reports, protocols, clinical practice guidelines, theses, dissertations, and final academic projects were considered. Letters, editorials, articles with incomplete data (conference proceedings, pilot projects), review articles, and studies not available in full were excluded. Articles addressing humanoid robots in clinical practice, with complete text, detailed scientific methodology, and appropriate quality were included. Selected studies were exported to the Rayyan QCRI reference manager software, where duplicate studies were removed. The study selection proceeded in two stages: 1) title and abstract screening by two reviewers, selecting all eligible studies within the Rayyan manager; 2) full-text reading of the studies selected in the previous phase by the same reviewers. In cases of disagreement, a third reviewer conducted an evaluation.

*Data extraction.* Data were extracted by two reviewers using a spreadsheet created by the authors in Excel®. Extracted information included bibliographic details - title, author, publication year, Digital Object Identifier (DOI); language; institution, city, and country of study origin; sample size; study population characteristics; study objectives; methods, and main results; the role of the robots and type of care provided. The selected studies were categorized according to the nature of the care provided by HRs into the following categories: social interaction, physical rehabilitation, and health information dissemination. Social interaction included care in the form of therapeutic interventions aimed at promoting well-being, facilitating treatment, and improving health outcomes through effective communication, empathy, emotional support, and education. Studies addressing this type of intervention were classified under social interaction care.^19^ Those focused on coordinated interventions designed to reduce disability and improve the quality of life of individuals with physical, mental, or emotional limitations were classified under physical rehabilitation care.^20^ Studies centered on disseminating health-related information to improve service quality and patient knowledge were categorized as health information dissemination care.^21^

*Data synthesis.* Descriptive methods were used to present the data, which were expressed through a narrative approach and explanatory tables, detailed in the results section.

## Results

**Figure 1:**
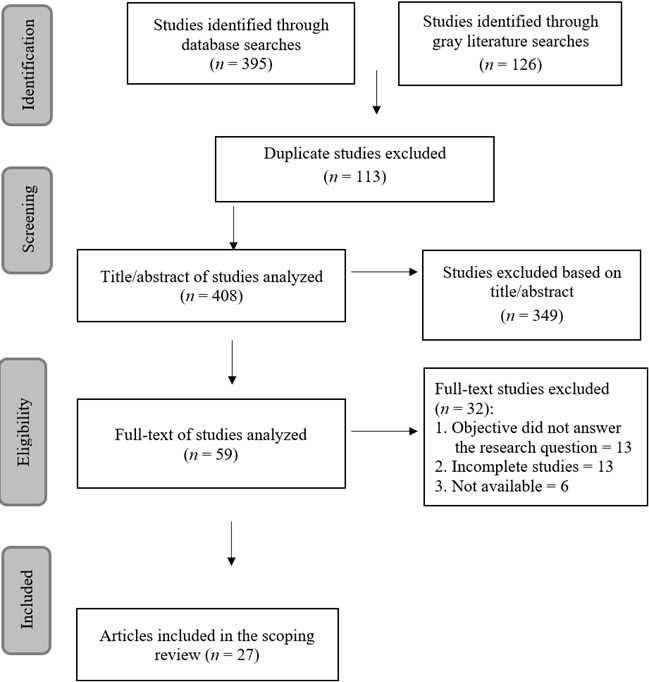
PRISMA flowchart of the results from search number 2, conducted in November 2023. Londrina, PR, Brazil, 2024

In the second search, conducted in November 2023, a total of 521 studies relevant to the research were initially identified. After a rigorous exclusion process, 494 studies that did not meet the predefined criteria were removed, resulting in 27 selected publications that provided direct answers to the research question. These results are detailed in Figure 1.

Of the studies included in the analysis, it was observed that 31.0% were conducted in Europe, with a notable concentration in France (*n* = 4), and another 31.0% in Asia. Within the Asian region, Japan stands out with a representation of 55.6% (*n* = 6), while 20.7% of the research was conducted in Canada (*n* = 5), and only one study was identified from Brazil.

The publication period of the studies spans from 2016 to 2023, with the majority (69.0%) being published between 2019 and 2021.^(2, 22-35)^ The predominant language in the studies was English, present in 96.5% of the publications. In terms of the locations where the studies were conducted, 13 occurred in hospital environments^(2, 22-24, 27, 28, 30, 31, 35-37, 39, 40)^ and nine in outpatient clinics.^(26,29,33, 40-45)^ Furthermore, three studies^25^^,^^26^^,^^30^ were carried out in long-term care facilities, three in university research centers,^32^^,^^34^^,^^48^ and one in a private residence.^49^ Two studies had two types of settings, outpatient and hospital.^30^^,^^40^

Regarding the type of population studied, ten of the studies were conducted with adults,^32^^,^^34^^,^^35^^,^^39^^,^^40^^,^^42^^,^^43^^,^^45^^,^^46^^,^^48^ eight involved children,^2^^,^^22^^,^^27^^,^^28^^,^^36^^,^^37^^,^^41^^,^^44^ seven focused on older adults,^25^^,^^26^^,^^30^^,^^31^^,^^33^^,^^43^^,^^49^ and four targeted healthcare providers.^24^^,^^27^^,^^31^^,^^43^ One study included a population of older adults, adults, and healthcare providers.^43^ In the selected studies, it was observed that the humanoid robots (HR) were developed in both adult and child forms,^25^^,^^47^ and even covered in fur to effectively interact with each type of patient.^48^

Regarding the areas of care addressed, social interaction was the most prevalent, representing 62.1% of the studies, followed by physical rehabilitation (24.1%) and health information (10.3%). These data are presented in Tables 1, 2, and 3 according to the chronological order of the life cycle.

Regarding the studies addressing social interaction, the majority were conducted with pediatric populations, with older adults, and to assist healthcare providers as an adjunct tool, optimizing treatment (Table 1).

**Table 1:** Characterization of the studies included in the scoping review related to care categorized as social interaction performed by humanoid robots

Author and year of publication	Study development location	Characteristics of the population	Robot's Role
Alemi *et al.*, 2016^36^	Mahak and Markaz-e-Tebi-Koodakan Hospitals (Tehran, Iran)	Children diagnosed with cancer aged 7 to 12 years.	The robot assumed the role of a therapist's assistant, with the goal of instructing children about their disease and symptoms, empathizing with them, and providing a space for them to express their fears and concerns.
Ali *et al.*, 2021^22^	Stollery Children's Hospital ED (Edmonton, Alberta, Canada)	Children undergoing intravenous (IV) puncture.	The robot performed distraction therapy during IV, aiming to reduce suffering and pain in children.
Farrier, Pearson & Beran, 2020^27^	Alberta Children's Hospital (Alberta, Canada)	Children aged 2 to 15 years.	The robot interacted with children using strategies to distract them, such as telling jokes, playing music, and performing dances. Additionally, the robot used techniques such as encouragement, guided imagination, and breathing exercises.
Lee-Krueger *et al.*, 2021^2^	Tertiary pediatric hospital (Alberta, Canada)	Children undergoing intravenous puncture.	The robot taught deep breathing as a coping strategy for pain and fear in children.
Meghdari *et al.*, 2018^37^	Ali Asghar and Mahak Hospitals (Tehran, Iran)	Children aged 5 to 12 years diagnosed with cancer.	The robot interacted with children with cancer through storytelling, aiming to reduce their suffering.
Meyns *et al.*, 2019^28^	Primary school and University Hospital of Ghent (Ghent, Belgium)	Children with normal development and with oncological changes.	The robot engaged children in physical activity exercises. It also performed pre-programmed dances with music.
Uluer *et al.*, 2023^41^	Clinic (Istanbul, Turkey)	Children with hearing impairment.	The robot interacted in a personalized way during audiometry tests and rehabilitation, based on the children's needs, with the goal of improving their social connection, communication, and overall well-being.
Sarabia *et al.*, 2018^39^	Chelsea and Westminster Hospital (London, England)	Adults hospitalized for surgery, infections, or dementia.	The robot conversed, told jokes, played music, danced, and exercised with the patients.
Yoshida *et al.*, 2022^42^	Keio University School of Medicine (Tokyo, Japan)	Adults with social anxiety disorder (SAD) and autism spectrum disorder (ASD).	The CommU robot (Communication + U - You) allowed patients to communicate through it, avoiding eye contact with others, reducing anxiety, and increasing confidence in speaking.
Cohen *et al.*, 2017^47^	Department of Adult Psychiatry at the University Hospital of Montpellier (Paris, France)	Adults with schizophrenia.	The robot interacted using non-verbal signals, demonstrating facial expressions, hand gestures, body postures, and eye direction.
Chen *et al.*, 2020^25^	Long-term care institutions (Hong Kong, China).	Older adults with dementia.	The robot, with a childlike appearance, performed interactions by speaking, singing, and shaking its head in response to various stimuli (e.g., knocks, tremors, and spoken words).
Chen *et al.*, 2020^26^	UAge Day Care Center (Taipei, Taiwan).	Older adults with neurocognitive diseases.	During group sessions, the robot stimulated the exchange of previous life experiences and the learning patterns of participants to improve interaction and communication skills.
Lee *et al.*, 2023^48^	Nursing homes (Seoul, South Korea)	Older adults from low-income backgrounds.	The Hyodol SAHR's reminded users to eat meals, take medications, attend medical appointments, and participate in social interactions. It encouraged guided exercises, including body movements and simple stretches, as well as recreational activities through popular songs and quiz games.
Tanioka *et al.*, 2021^30^	Psychiatric hospital and nursing home. (Tokushima, Japan)	Older adults with schizophrenia and dementia.	The robot conversed with older adults, creating a sense of connection and joy, indicating its potential to facilitate positive emotions in individuals with schizophrenia and dementia.
Beran *et al.*, 2020^23^	Alberta Children´s Hospital (Alberta, Canada).	Healthcare providers.	The MedI® (Medicine and Engineering Designing Intelligence) robot interacted with children at the bedside, aligning with the functions and responsibilities of healthcare providers.
Beran *et al.*, 2021^24^	Alberta Children´s Hospital (Alberta, Canada).	Healthcare providers.	The MedI® robot provided emotional support and engaged children in therapeutic activities. The robot was incorporated into the daily practices of healthcare providers.
Tanioka, 2019^29^	Rehabilitation facilities for older adults. (Okayama, Japan)	Occupational therapists and nurses.	The robot acted as a mediator in the relationship between the older adult and the occupational therapist, facilitating communication and interaction.

In the context of physical rehabilitation, it can be seen that most studies investigated the use of robots as an auxiliary tool in the practice of physical exercise, with good interaction with patients (Table 2).

**Table 2:** Characterization of the studies included in the scoping review related to care categorized as physical rehabilitation actions performed by humanoid robots

Author and year of publication	Study development location	Characteristics of the population	Robot’s Role
Blanchard *et al.*, 2022^45^	Rehabilitation centers in Brittany (Brittany, France)	Adults with low back pain, incomplete spinal cord injury, and nonspecific chronic low back pain.	The robot supervised and guided participants in performing stretching exercises for chronic lower back pain.
Platz *et al.*, 2023^40^	University Medical Center of Greifswald and Greifswald Clinic (Greifswald, Germany)	Adults post-stroke with severe hemiparesis.	The robot welcomed the patient individually and explained the therapeutic goal, the prescribed therapy, and how the individual training tasks worked. It provided audiovisual instructions through photos and videos, offered feedback according to the type of therapy, and provided breaks as needed.
Feingold-Polak *et al.,* 2021^46^	"Adi Negev" Rehabilitation Center (Jerusalem, Israel)	Adults post-stroke.	The robot interacted with participants, providing feedback, displaying images for correction, and motivating them to continue their rehabilitation exercises.
Garcia, 2019^32^	Technology Center of the Federal University of Santa Maria - RS (Santa Maria, Brazil)	Individuals over 18 years of age.	The robot served as a motivational tool for physical exercises and their psychological aspects through interactions with participants. It aimed to encourage and engage individuals during exercise practice.
Aubin *et al.*, 2021^34^	Montpellier University (Montpellier, France)	Adults with schizophrenia.	The NAO robot (Japanese word = simple) acted in two ways. It synchronized movements with the patients, and at other times, it performed the movement, and the patient observed to replicate it later.
Ujike *et al.*, 2019^31^	Mifune hospital (Kagawa, Japan)	Older adults with schizophrenia and decreased physical function who needed to move in a wheelchair.	The robot intentionally communicated with the patient, asking questions about their knowledge and experiences, and the patients answered the questions. Patients actively participated in the Care Prevention Exercises using Pepper-CPGE and followed its instructions while demonstrating calisthenics exercises.
Tanioka *et al.*, 2020^33^	Facilities for the elderly (Japan)	Older adult, 69 years of age, with schizophrenia.	The robot assisted in performing Care Prevention Gymnastics Exercises (Pepper-CPGE) through an audio device, lasting 3 minutes.

Regarding health information, few studies were identified, and all applied robots as a tool for transmitting important health information, with a primary focus on patients (Table 3).

**Table 3:** Characterization of the studies included in the scoping review related to care categorized as health information dissemination performed by humanoid robots

Author and year of publication	Study development location	Characteristics of the population	Robot’s Role
Al-Taee *et al.*, 2016^44^	King's College Hospital waiting room (London, England)	Children with type I diabetes mellitus (DM).	The robot provided guidance on managing DM, including information on blood sugar monitoring, insulin administration, and maintaining a healthy lifestyle. It supported the education of children on DM self-care and promoted healthy habits.
Stoevesandt *et al.*, 2021^35^	Halle University Hospital (Saale, Germany)	Adults undergoing elective magnetic resonance imaging examination.	The robot interacted with patients and conveyed information about the magnetic resonance imaging examination through voice commands and body language.
Blavette *et al.*, 2022^43^	Memory Clinic of the Broca Hospital and Rehabilitation Clinic of the Vaugirard Hospital (Paris, France)	Older adults, caregivers, and healthcare providers.	The robot communicated information through both verbal and non-verbal behaviors, presenting COVID-19 precautions based on official information.

## Discussion

The global health of the population is influenced by various factors, including technological advancements and the impact of the Fourth Industrial Revolution,^49^ which began in the current century. Robotics is part of this context, and the present study provides a comprehensive review, highlighting its predominant application in healthcare in European and Asian countries.^50^ The Fourth Industrial Revolution, as described by Klaus Schwab in 2016, is transforming the healthcare sector through technologies like artificial intelligence, biotechnology, and the Internet of Things. These innovations are changing the way healthcare services are provided and managed, increasing efficiency and personalized access to care. The integration of these technologies could create a more ethical, inclusive, and sustainable future. Experts in machine learning and data ethics are essential to ensure that these advancements improve global quality of life and safety.^51^

Isaac Asimov, one of the pioneers of science fiction, envisioned as early as the 1940s a future in which robots and humans coexisted for the progress of humanity. His stories reflected on the ethical and social implications of robotics, emphasizing the safe integration of robots into society, from household tasks to complex functions. Asimov addressed moral dilemmas, exploring the responsibility of intelligent robots. He predicted that, with technological advancements, robots could develop a deep understanding of human emotions and become valuable allies.^52^

The new healthcare model created by the Fourth Industrial Revolution was presented at the Hannover Fair in 2011 in Germany, involving the automation of industrial processes, merging digital technology, artificial intelligence, and connectivity.^53^ From this, humanoid robots emerged, an advanced form of automation capable of performing complex tasks that were previously carried out exclusively by human workers.^54^ In the present study, it was identified that in the healthcare sector, robots use gestures, speak, perform facial recognition, move, and, in general, promote social interaction to assist patients, aiming to provide care and achieve measurable progress in recovery, rehabilitation, learning, and well-being. Currently, healthcare companies are innovating in the production and development of robots to meet the universal technological demands of this field.^35^ As identified in this study, research in robotics in Europe is extensive and distributed across the region. Different European countries have made significant contributions to the advancement of robotics in various areas, such as industrial robotics, medical robotics, service robotics, and autonomous robotics.^55^ Germany is known for its strong engineering and automation industry, with German companies playing a crucial role in the development of industrial robots and automation systems.^56^^,^^57^ Collaboration between research institutions, universities, and industries across Europe is common, supporting the ongoing development and advancement of robotics in the region.^58^ Other studies occurred in the Asian continent, certainly reinforced by Japan, which has a long history of creating industrial robots and is a leader in service robotics. Japanese companies have developed advanced humanoid robots for various applications, including care and entertainment for older adults.^59^ The Japanese government strongly supports the development of care robots to address the challenges of an aging population and the shortage of healthcare providers.^60^

The COVID-19 pandemic increased visibility and interest in the use of AI technologies and robots on the frontlines of healthcare, justifying the rise in publications on the topic since 2016, with a surge after 2019. During this period, robots were introduced into the medical sector to perform support tasks, while more complex activities remained the responsibility of professionals. In addition to minimizing contact between infected patients and healthcare providers, research advanced in the teleoperation of robots, allowing remote control for monitoring vital signs and other medical procedures.^61^^-^^64^ Regarding the type of care found in the results of this review, robot-patient interactions were highlighted across various age groups; these interactions impact engagement and adherence to the proposed treatment,^37^ as humanoid robots have the ability to act as agents of therapeutic distraction during medical procedures, alleviating anxiety and discomfort associated with clinical interventions.^65^

Health information care was also identified, where the robot continued interacting with the human, but the primary goal was to transfer health information consistently and accurately, following established protocols and guidelines, ensuring that all patients received the same quality information, regardless of when they interacted with the robot.^43^ Through the studies analyzed, it was found that one of the populations studied was children. Research in the pediatric area can be justified by the creativity and imagination of this audience: some findings demonstrated the use of humanoid robots in educating children with specific health problems or special needs, developing childhood skills, promoting healthy behaviors, and providing comfort during medical procedures.^66^^,^^67^

By providing entertainment and playful interaction, these robots can modulate pain perception and facilitate the completion of medical procedures, making the experience more tolerable, especially for children.^68^ In this age group, the human-robot interaction can also stimulate their emotional and social development, offering companionship and emotional support, therefore reducing the feeling of fear.^68^ The ability of these robots to express emotions and respond sensitively to children's emotions can contribute to the effectiveness of treatment, prognosis, and, above all, quality of life.^28^

The interest in the adult population can be demonstrated by the ability to personalize treatment, which involves adapting instructions to meet the specific needs of patients, providing the best outcomes in rehabilitation and medical care. As mentioned earlier, this began in 1990 with advancements in the field of robotic surgery.^69^ Some studies investigated rehabilitation care using humanoid robots to assess their effectiveness in improving physical function in adults after injuries, strokes, or surgeries.^40^ In the literature, studies^71^^-^^74^ investigated parameters such as muscle strength, range of motion, and motor coordination. The use of robots in upper limb rehabilitation has shown promising results in improving musculoskeletal functions, including strength, sensation, perception, vibration, muscle development, spasticity reduction, flexibility, and range of motion.^70^^-^^73^ These findings present a relatively small sample, which may limit the representativeness of the results in relation to the broader population of studied diagnoses. Moreover, the detailed characterization of participant diversity, including variables like age, gender, and disease severity, is not always explored, thus influencing the generalization of results, as different clinical profiles may respond differently to the intervention.

The scientific interest in rehabilitation with HRs is multifaceted and spans various research areas; in general, researchers are interested in determining the effectiveness of humanoid robots as rehabilitation tools compared to traditional methods.^31^ There is an interest in developing robotic systems that can be adapted to the specific needs of each patient, personalizing treatment to the individual capabilities and goals of each one.^74^ Overall, HRs offer an innovative and promising approach to patient rehabilitation, complementing the work of human therapists and offering unique benefits that may help patients achieve their rehabilitation goals more effectively.^40^

On the other hand, the connection of AI with robotics provides means to meet some of the care needs of other population groups, such as older adults. Concerning older adults, international literature confirms that the population aged over 60 is growing at an accelerated rate compared to other age groups in developed countries and even those in development, such as Brazil.^75^ In Japan, the aging population is inversely proportional to birth rates: the older adult population is growing significantly. As a result, research focusing on analyzing ways to meet the demands and vulnerabilities of older patients stands out,^29^ providing assistance in daily tasks such as mobility, personal hygiene, and health monitoring. Robots can be equipped with sensors to monitor signals of interest in older adults, such as blood pressure, heart rate, and blood glucose levels. This allows for early detection of health problems and a rapid response in case of emergencies.^76^^,^^77^ One point of attention is ensuring that systems operate safely and reliably, especially in health-related care. Failures in the system can lead to unexpected or inappropriate movements, putting the patient's safety at risk.

The literature presents the application of robotics in assisting older adults with Alzheimer's disease, which, in its early stages, can provide significant benefits, particularly by promoting autonomy, stimulating cognitive functions, and offering emotional support, contributing to the improvement of these individuals' quality of life. However, the implementation of these technologies is not without obstacles, including ethical issues, privacy concerns, and the need for high-quality training data to ensure effective human-robot interaction.^83^

HR are also known as bipedal robots and are used in less complex care to minimize the overload on the healthcare system, prioritizing the care provided by nursing professionals for more complex situations.^43^ The literature shows HRs in long-term care facilities, providing company to older adults and those requiring palliative care;^78^ thus, in addition to promoting a better quality of care, robots allow the healthcare team to focus on other tasks for patients.^68^ The natural social isolation of older adults, caused by health conditions, reduced mobility, or lack of family support, aggravates clinical complications and functional decline, increasing vulnerability to new diseases. In this context, HRs have shown potential to act in rehabilitation, as well as provide company and social interaction, helping to reduce isolation, promote emotional well-being, and offer entertainment, especially in cases of mental health issues such as depression and anxiety.^26^^,^^48^ Another population studied includes healthcare providers.^29^^,^^27^^,^^24^^,^^43^ Various factors such as stress, occupational risks, dual work shifts, and low wages can lead to burnout, impairing their health, quality of life, and even their performance in delivering care.^45^ Therefore, findings support that robots do not aim to replace these healthcare providers; their role will always be to provide true human care, and with the help of robots, they can focus on actions requiring human relationships and more complex treatments.^79^

It is worth noting that, despite the availability of numerous training programs for those wishing to work in this field, the demand for qualified professionals continues to rise. This is due to both the accelerated aging of the population and the increasing demand for general care; thus, there is a trend toward a shortage of labor in healthcare.^80^ However, the insertion of humanoid robots in the care process may allow healthcare providers to focus on care that must exclusively be provided by them.

HR can be placed in strategic locations such as hospitals, clinics, or healthcare centers, where they can provide health information in an accessible manner to a wide range of people, regardless of their geographical location or technological skills. Unlike healthcare providers, who have work hour limitations, bipedal robots can be available to provide health information at any time of the day, thus increasing accessibility to healthcare.^81^ Additionally, humanoid robots can help reduce the workload of healthcare providers, allowing them to concentrate on more complex and demanding cases.^82^

The conclusion of this study is that, although the use of humanoid robots in healthcare is increasing, particularly in hospitals, there is still a strong need for better integration between these machines and healthcare providers. The focus on social interaction in many studies suggests an opportunity to improve the patient experience, but also highlights the need to further investigate physical rehabilitation and information sharing. Thus, future research should aim to create protocols that successfully combine the abilities of humanoid robots with the knowledge of healthcare providers, ensuring that technology not only supports but also improves the quality of patient care and fosters a more effective team-based work environment, always within the ethical principles of healthcare practice.

In terms of study limitations, it can be considered that the clarity of the topic influenced the selection of studies, affecting the robustness of the data and the consistency of the established parameters. The limited sample of studies impedes the generalization of the results. Moreover, the inclusion of research in early stages, combined with restricted access to databases and scientific publications, compromised the breadth and depth of the analysis. Despite these limitations, the results provide an important foundation for future investigations, with potential to guide healthcare practices and policies. The study seeks to deeply integrate technology into healthcare, offering insights to improve service quality, as several countries are becoming potential hubs for robotics innovation in healthcare.
